# The role of soluble adenylyl cyclase in sensing and regulating intracellular pH

**DOI:** 10.1007/s00424-024-02952-x

**Published:** 2024-04-06

**Authors:** Hang Lam Li, Arthur Verhoeven, Ronald Oude Elferink

**Affiliations:** Tytgat Institute for Liver and Intestinal Research, Research Institute AGEM, Amsterdam UMC, Meibergdreef 69-71, 1105BK, Amsterdam, the Netherlands

**Keywords:** Soluble adenylyl cyclase, Intracellular pH, tmAC

## Abstract

Soluble adenylyl cyclase (sAC) differs from transmembrane adenylyl cyclases (tmAC) in many aspects. In particular, the activity of sAC is not regulated by G-proteins but by the prevailing bicarbonate concentrations inside cells. Therefore, sAC serves as an exquisite intracellular pH sensor, with the capacity to translate pH changes into the regulation of localization and/or activity of cellular proteins involved in pH homeostasis. In this review, we provide an overview of literature describing the regulation of sAC activity by bicarbonate, pinpointing the importance of compartmentalization of intracellular cAMP signaling cascades. In addition, examples of processes involving proton and bicarbonate transport in different cell types, in which sAC plays an important regulatory role, were described in detail.

## Introduction

Adenylyl cyclases convert ATP into cyclic AMP, one of the most important intracellular second messengers. The family of mammalian adenylyl cyclases comprises ten members, nine of which are transmembrane adenylyl cyclases (tmAC) encoded by *ADCY1* to *ADCY9* genes. tmACs are localized at the inner surface of the plasma membrane, and their activity is modulated by G-proteins which interact with G-protein coupled receptors. G-proteins either stimulate (Gα_s_) or inhibit (Gα_i_) the cAMP-producing activity of tmACs. A more recently discovered adenylyl cyclase is the tenth member of the family, encoded by *ADCY10*. The gene product of *ADCY10*, soluble adenylyl cyclase (sAC), is fundamentally different from the other members of the family and is related to the most ancient forms of adenylyl cyclase present already in archaebacteria [56]. sAC differs in many aspects from the other mammalian family members: it is not an integral membrane protein, and it is not localized at the plasma membrane but in several intracellular locations. Hence, sAC is not activated by G-proteins but its activity is mainly regulated by the prevailing bicarbonate concentrations. Litvin et al. [34] showed that the most active isoform of human sAC is about 30-fold more active at a physiological bicarbonate concentration of 10 mM. Indeed, titration of the enzyme with HCO_3_^−^ concentrations displays a steep dependence in the physiological range. Hence, this renders sAC an exquisite intracellular pH sensor. Intracellular bicarbonate concentrations are in direct equilibrium with intracellular pH; the ubiquitous presence of carbonic anhydrases (catalyzing the reversible hydration of CO_2_) with the highest molecular activity of any known enzyme, (about 36 × 10^6^ molecules/min) ensures rapid equilibrium between CO_2_ and HCO_3_^−^ [40]. Besides functioning as a pH sensor, sAC also functions as a metabolic sensor: in contrast to the tmACs, which have a low K_m_ for ATP, sAC has a high K_m_ for ATP within the range of the prevailing intracellular concentrations. As a consequence, sAC activity is also determined by changes in the cellular energy state. In addition, it was recently reported by Laudette et al. [31] that sAC can be palmitoylated in cardiomyocytes cultured in the presence of palmitate. This palmitoylation activates the enzyme with subsequent cAMP-mediated activation of EPAC1 (exchange factor directly activated by cAMP 1) and subsequent mitochondrial oxidative stress and dysfunction leading to cardiomyocyte cell death. This did not occur in Epac-deficient cells, suggesting that the sAC/cAMP/EPAC1 axis plays a role. The latter aspect of sAC falls, however, beyond the scope of this review.

## cAMP signaling in microdomains

Any interpretation of the importance of intracellular cAMP levels should take into account the location where cAMP is produced. Local cAMP levels are determined by the local synthesis but also by the local breakdown of cAMP. Thus, the compartmentalization of synthesis is determined by protein interaction with other components in the signaling cascade. A-kinase anchoring proteins (AKAPs) are well known for their ability to scaffold protein kinase A and components upstream and downstream of cAMP production, including G protein-coupled receptors, cAMP-dependent Rap-exchange factors, and phosphodiesterases. Specific adenylyl cyclase (AC) isoforms have also been identified as components of AKAP complexes. Furthermore, the organization of tmACs in lipid rafts organizes these enzymes in close proximity to their up- and downstream members of the signaling cascade. For sAC, this organized localization has been investigated much less extensively, but it is clear that sAC is highly localized in organellar structures such as mitochondria, endosomes, and nuclei as well as at the cytoskeleton and cilia. Apart from localized synthesis, also the localized breakdown of cAMP by specifically localized phosphodiesterases has a major effect on cAMP signaling [38, 45]. This strict and specific localization is highlighted by the fact that different adenylyl cyclases can have opposite functions. An example of this is the reported opposite regulation of glycogenolysis by tmACs and sAC [6]. It is well established that forskolin, an activator of tmACs, stimulates glycogenolysis, but LRE1, a specific inhibitor of sAC, also stimulates glycogenolysis (forskolin has no stimulatory effect on sAC). The same phenomenon was also observed for the pro- vs. anti-apoptotic effect of cAMP derived from sAC and from tmACs, respectively [11]. A third example of simultaneous but independent regulatory mechanisms by cAMP from tmACs vs. sAC has been reported for melanosomal pH and pigmentation in melanocytes [67]. Hence, information on the role of generated cAMP in intracellular regulation should ideally be accompanied by the cellular site of synthesis and breakdown.

## Expression and inhibition of sAC in various cells and tissues

Originally it was thought that expression of sAC is restricted to the testis and more specifically to the sperm cells [8]. This is understandable because expression of the *ADCY10* gene is by far the highest in the testis. Nevertheless, expression of this enzyme even in testes appeared relatively low; isolation of sAC protein required 950 rat testes to obtain enough material for characterization and a partial protein sequence [8].

The original isolation and cloning of mammalian (rat) sAC by Buck et al. [8] describe a gene product encoding a “truncated” protein of 48 kD with cAMP-forming activity from rat testis that is derived from a cDNA encoding a much larger protein of 187 kD. The amino acid sequence of the truncated version of the protein did contain two adenylyl cyclase activity domains. The authors demonstrated that expression of the full-length cDNA produced a protein with about ten-fold lower activity. The same group later revealed that the reduced activity of the full-length protein is likely caused by an autoinhibitory sequence C-terminal to the second activity domain [10]. Clearly, the single *Adcy10* gene gives rise to multiple isoforms with very different characteristics. Geng et al. [20] analyzed immunoreactive sAC polypeptide by western blotting in various cell lines (HEK-293, OKP, NRK, and LLCPK1, Caco-2, and primary osteoblasts). This revealed two bands at ~ 190 kD and ~ 80 kD, with the 80-kDa band being the dominant polypeptide. Recently, Go et al. [23] described an isoform of sAC with similar molecular weight in cholangiocytes that is transported into the endoplasmic reticulum and undergoes glycosylation. This protein is rapidly secreted in extracellular vesicles. It remains to be determined whether the latter two studies observed the same isoform.

RT-PCR analysis later showed that *ADCY10* expression occurs in virtually all cells albeit at (sometimes very) low levels that may escape protein detection. Other tissues besides the testes with detectable sAC protein levels are the liver, heart, and various cancers such as lung, breast, and liver cancer [65] as well as cell types such as epididymal clear cells, kidney tubular epithelial cells, bile duct epithelial cells, and fibroblasts [25, 39, 42, 57].

### The use of first-generation sAC inhibitors should be avoided

Due to the low expression levels of sAC in many cell types, much work has been performed with inhibitors of sAC. In this respect, it must be mentioned that the first-generation inhibitors, 2-hydroxyestradiol, and KH7, are not very specific. KH7 has been demonstrated by various groups as a direct and strong inhibitor of complex I of the respiratory chain. Lark et al. [30] assayed Complex I activity in isolated mitochondria and provided compelling evidence that KH7 is a direct inhibitor of complex I with an IC_50_ of 4 nM. Importantly, the inhibition could not be relieved by the addition of 8Br-cAMP to the incubation strongly suggesting that it was not mediated by the sAC/cAMP/(PKA) axis. They furthermore observed that the other sAC inhibitor, 2-hydroxyestradiol, a naturally occurring estrogen metabolite, spontaneously generates high rates of H_2_O_2_, which can have serious side effects. Moreover, De Rasmo et al. [15] found that in fibroblasts, KH7 induces rapid degradation of several Complex I subunits by mitochondrial protease or by the proteasome. The latter effect of KH7 appeared, however, to be rescued by 8Br-cAMP. The second-generation sAC inhibitor, LRE1, is much more specific [49]. A set of third-generation inhibitors with nanomolar EC_50_ is now under investigation and may be developed for use as a male contraceptive drug [4].

## sAC as an intracellular pH sensor and regulator

The group of Levin and Buck was the first to coin sAC as an intracellular pH sensor on the basis of its steep induction of cAMP production by physiological concentrations of bicarbonate [12]. Since then, this characteristic has been confirmed by a number of other groups studying the enzyme. As indicated in the introduction, sAC displays homology with adenylyl cyclases in lower organisms (such as bacteria), and Kobayashi et al. [29] demonstrated that the bicarbonate-activating property of sAC was already present in adenylyl cyclases from bacteria such as Chloroflexus bacteria. Hence, bicarbonate activation of sAC is an ancient evolutionary property.

Like all adenylyl cyclases, sAC has two catalytic domains (C1 and C2), both necessary for activity. Analysis of the crystal structure of sAC [28] revealed that the stimulation of sAC activity by bicarbonate involves a small insertion in the C2 domain that converts it into a binding site for bicarbonate and precludes binding of the tmAC-activating diterpene forskolin, which explains the insensitivity of sAC towards forskolin. Bicarbonate binding to this site appears to increase activity by facilitating the open/closed transition that is assumed to occur during catalysis [27]. Some isoforms of sAC lack the C1 domain and would therefore not be active. It has been hypothesized, however, that these isoforms may dimerize with other cyclases that would donate the missing C1 sequence [13].

## sAC functions as an intracellular pH sensor by modulating ion transporters

With the immediate implication of sAC as a pH sensor in response to intracellular bicarbonate concentration, a considerable number of studies were conducted to detail the role of sAC-mediated signaling in cellular pH sensing and pH regulation. Due to the plasticity of electrolyte transporter expression in various tissues, it is more appropriate to discuss sAC-dependent pH regulation on a cell and tissue basis.

### Fibroblasts

Mardones et al. [39] confirmed the bicarbonate-induced cyclase activity in intact murine fibroblasts and showed that intracellular alkalinization (i.e., increase in bicarbonate concentration) not only induced cAMP production by sAC but also dramatically increased its gene transcription (> four-fold). This could be demonstrated by the comparison of fibroblasts from *Slc4a2*^+*/*+^ and *Slc4a2*^*−/−*^ mice. *SLC4A2* is a chloride/bicarbonate exchanger (AE2) that under physiological conditions mediates the excretion of HCO_3_^−^; its absence leads to accumulation of HCO_3_^−^ with a concomitant alkalinization by 0.2 pH units. Interestingly, this alkalinization was also accompanied by a dramatic reduction in the expression of carbonic anhydrase 2 (CA2), the most abundant carbonic anhydrase, responsible for the rapid and reversible hydration of CO_2_. Paradoxically, acute inhibition of sAC activity with KH7 also led to decreased CA2 expression. It was hypothesized that this might be due to an incoherent feed-forward regulation of CA2 by sAC-derived cAMP [39]. It could also be that the effect of KH7 was in this case caused by unspecific side effects of the inhibitor as described above.

### Hepatocytes

Interestingly, a recent publication by Liu et al. [35] demonstrated that sAC activity is regulated by yet another mechanism involving the binding and activation of sAC by lactate. Incubation of the enzyme with increasing concentrations of lactate stimulated sAC activity more than five-fold with an EC_50_ of around 24 mM. This phenomenon may sound counterintuitive because lactate production is accompanied by intra-and extracellular acidification whereas sAC was found to be stimulated by alkaline conditions. However, crystallization of the protein in the presence of acetate (not active in stimulating sAC activity), bicarbonate (the prototypic activator), and lactate revealed that lactate binds at the same site as bicarbonate. Hence, in the presence of high lactate concentrations, the lack of HCO_3_^−^ is compensated by the binding of lactate. Normal lactate concentrations are in the range of 1.5–3 mM in the blood and tissues of healthy individuals, but under inflammatory conditions and in tumor tissue, these can rise to 40 mM [46].

### Sperm cells

The highest sAC expression is in the testis and, indeed, bicarbonate homeostasis plays a crucial role in sperm maturation and capacitation [9]. The lumen of the seminiferous tubules of rat testes presents a pH of around 7.31 and 20 mM HCO_3_^−^. In the caput (proximal) region of the epididymis, the pH as well as the concentration of HCO_3_^−^ decreases (pH 6.6 and 2.7 mM HCO_3_^−^), whereas in the cauda (distal) region of epididymis and vas deferens, it slightly rises again (pH 6.85 and 6.7 mM HCO_3_^−^) [32]. In the epithelial cells of all these compartments, the electroneutral chloride/bicarbonate exchanger AE2 (*SLC4A2*) is expressed. Being the most abundant “housekeeping” chloride/bicarbonate exchanger, it functions as an acid loader in most tissue cells. Indeed, studies in *Slc4a2*^*−/−*^ mice demonstrated that spermiogenesis is already impaired in the round spermatid stage, and no mature spermatozoa are present with complete male infertility as a consequence [41]. However, at this stage of spermiogenesis, sAC does not yet play a crucial role, since Adcy10^−/−^ mice have complete spermiogenesis. However, sperm motility and capacitation are strongly impaired in these mice, resulting in male infertility [18]. Hence, sAC plays a crucial role at a later stage in the spermiogenesis, where intra- and extracellular bicarbonate concentrations determine the motility of spermatozoa.

In sperm motility and capacitation, intracellular alkalinization is associated with three steps. First, elevations of intracellular pH initiate and modulate flagellar motility. Second, the development of mammalian sperm fertility occurs within the female reproductive tract through the process of capacitation and is associated with pH elevation. Sperms fail to capacitate when alkalinization is prevented. Finally, sperms of many animal species must complete the acrosome reaction (AR). In mammals, the AR is controlled by adhesive contacts between sperm and the egg’s zona pellucida (ZP). The ZP-activated signal transduction pathway includes an elevation of intracellular pH.

Regarding flagellar motility, an important finding in this regard was the study by Wang et al. [62] who showed that there is a complex interaction between the sperm-specific Na^+^/H^+^ exchanger (sNHE) and sAC. Localized in the flagellum of the sperm, sNHE is a member of the NHE family and is responsible for the sodium-driven extrusion of protons, leading to intracellular alkalinization and elevation of bicarbonate concentrations. Wang et al. observed that disruption of the sNHE gene in the mouse led to complete male infertility. Sperm cells from these mice completely lacked the full-length sAC protein but left the levels of the much more abundant truncated isoform of sAC intact. They also showed that sNHE and sAC associate with each other and that the total adenylyl cyclase activity in the sNHE^−/−^ sperm cells was greatly diminished. This is an intriguing finding because the truncated sAC protein (of which the levels are normal) is about ten-fold more active than the full-length sAC. Thus, a complex regulation of sAC isoform activity occurs in conjunction with sNHE. The authors hypothesized that as CO_2_ diffuses into the cell and equilibrates into H^+^ and HCO_3_^−^, sNHE may exchange intracellular H^+^ for extracellular Na^+^ to increase intracellular bicarbonate levels.

More recent data from Windler et al. [64] establish sNHE (*SLC9C1*) as a phylogenetic chimera that combines the Na^+^/H^+^ exchange mechanism of solute carriers with the gating mechanism of ion channels. Hyperpolarization of the sperm cell “opens” the Na^+^/H^+^ exchanger. The presence of cAMP (produced by the associated sAC) reduces the hyperpolarization threshold of sNHE by about 20 mV thereby activating the Na^+^/H^+^ exchanger.

As for capacitation, it is also directed by hyperpolarization and intracellular alkalinization. When sperm enters the female reproductive tract, it encounters high concentrations of bicarbonate that is taken up by the cells by sodium-dependent bicarbonate co-transporters (NBC) [16]. Intracellular bicarbonate activates sAC (and this is accelerated by simultaneous Ca^2+^ entry [47]). Hyperpolarization is mediated mainly by SLO3, a K^+^ channel that is specifically expressed in sperm cells and activated by alkalinization and membrane voltage [55]. Mutations in this channel also lead to male infertility [36].

### Absorptive hypercalciuria and asthenozoospermia in humans with mutations in ADCY10

Of note, individuals with mutations in the *ADCY10* gene have been identified, although this is as yet limited to 59 patients worldwide [22]. Reed et al. [51] studied a group of patients with absorptive hypercalciuria displaying a familial transmission indicative of a genetic trait. A genetic linkage study suggested that the candidate gene lies on chromosome 1q23-24. They subsequently screened the relevant genes in this locus and found a gene with one or more mutations in 45 out of 80 patients. They sequenced the gene and found it to be 77% homologous to the rat *Adcy10* gene characterized by Buck et al. [8]. Hence, they connected the hypercalciuric phenotype to mutations in the *ADCY10* gene. Conversely, Visser et al. screened for mutations in a number of relevant genes in 30 patients with asthenozoospermia and 90 controls and found two patients with mutations in *ADCY10*. They could, however, not establish the impact of these mutations (one of them appeared to have normal bicarbonate-induced activity). All in all a relatively small group of patients has been described with mutations in *ADCY10* but in the majority of cases, it was not possible to demonstrate impaired function of the protein [7, 24, 33, 51, 60, 61, 63] (see supplemental figure in ref. [22] for a very recent overview of mutations). However, Akbari et al. [1] recently performed whole exome sequencing of two men (and several other family members) from a consanguineous family with both absorptive hypercalciuria and asthenozoospermia. This analysis revealed a homozygous 2 bp deletion in exon 11 (C2 domain) of the *ADCY10* gene leading to a truncation that is likely to disrupt activity. Analysis of the sperm of these males upon incubation with dibutyryl cAMP significantly improved motility for 30 min (but not longer). This convincingly demonstrated that these two individuals suffer from asthenozoospermia and hypercalciuria caused by a complete deficiency of sAC. Interestingly, several male heterozygotes of this family did not suffer from asthenozoospermia but did suffer from hypercalciuria. These observations may indicate that the sperm motility defect requires full absence of sAC whereas reduced activity may cause hypercalciuria. Indeed, the majority of individuals in whom *ADCY10* mutations were observed had hypercalciuria and only a few had asthenozoospermia.

### Cardiomyocytes

It is well established that ischemia/reperfusion causes apoptosis in cardiomyocytes. In a rat cardiomyocyte culture system, this process was studied by Appukuttan et al. [3], and they observed protein kinase A-dependent phosphorylation of Bax with subsequent translocation of Bax to mitochondria causing ROS formation. Based on experiments with the sAC inhibitor, KH7, they suggested a link between sAC and apoptosis via protein kinase A-dependent Bax phosphorylation at Thr(167) and its translocation to mitochondria during simulated ischemia, which subsequently caused mitochondrial oxygen radical formation followed by cytochrome *c* release and caspase-9 cleavage during simulated reperfusion. On the basis of the potential role of sAC-derived cAMP in this process, Espejo et al. [17] tested whether bicarbonate import via NBC and bicarbonate-dependent activation of sAC could play a role in this process. They found that KH7 reduced cardiac contractility and enhanced NBC-mediated bicarbonate import into cardiomyocytes. Hence, these experiments suggest a role of sAC in cardiac contractility. However, these studies were all performed with the non-specific sAC inhibitor KH7.

## Part 2: sAC regulates luminal pH by modulating the localization of V-ATPases

Vacuolar H^+^-ATPases (V-ATPase) comprise a family of ATP-dependent proton pumps, the subunits of which are organized into two domains: the 650kD cytosolic V_1_ domain and the 260kD membrane-embedded V_0_ domain. Upon ATP hydrolysis by the cytoplasmic V_1_ domain, protons are transported via the rotatory machinery in the integral V_0_ domain from the cytosol into the lumen of organelles or to the extracellular space [19]. V-ATPases are expressed ubiquitously in the endomembrane system, regulating the luminal pH of each compartment along the exocytic and endocytic pathways to maintain appropriate pH locally for specific physiologic processes.

### Epididymal epithelial cells

V-ATPase plays an important role in keeping the luminal pH of the epididymis relatively low to ensure sperm cells do not mature and remain non-motile until ejaculation; the subsequent neutralization by the alkaline seminal vesicle fluid then triggers the hypermotility of sperm cells. In the initial (proximal) segment, principal cells reabsorb HCO_3_^−^ (probably via NBC transport). In the cauda (distal) region of the epididymis and the vas deferens, clear cells further acidify the lumen through the action of V-ATPase in the apical membrane. The presence and activity of the V-ATPase need to be tightly regulated in order to maintain the proper pH set point. Of note, sAC is highly expressed in clear cells, and apical membrane accumulation of V-ATPase is triggered by a sAC-dependent rise in cAMP in response to alkaline luminal pH. Pastor-Soler et al. [42] investigated the role of bicarbonate in this regulation by perfusion with the carbonic anhydrase inhibitor, acetazolamide, at the cauda (distal) region of the epididymis. Upon this intervention, clear cells lost apical microvilli, and V-ATPase was present in intracellular vesicles. Under these conditions, the lumen had an alkaline pH. The addition of the cell-permeable cAMP analog, Cpt-cAMP, to the perfusate led to a rapid rearrangement of V-ATPase from intracellular vesicles to the apical membrane. The addition of the sAC inhibitor, 2-hydroxyestradiol, prevented this rearrangement, and the V-ATPase was present between intracellular vesicles and the microvilli [42]. The authors proposed that sAC is a crucial relay between prevailing bicarbonate concentrations and regulation of the amount of proton-secreting ATPase in the apical membrane. It was subsequently shown that the downstream effector of sAC-derived cAMP is PKA (and not EPAC). Thus, sAC senses bicarbonate and translates this into upregulation of V-ATPase activity in the apical membrane of the epithelial cells of the epididymis and vas deferens.

### Kidney epithelial cells

The kidney collecting duct is lined by transitional cells which can differentiate into principal cells (involved in Na^+^, K^+^, and H2O reabsorption) and intercalated cells (IC, involved in acid/base secretion). The latter cell type comes in two forms: one that acidifies the duct lumen (A-type IC) while the other alkalinizes (B-type IC) the duct lumen. Thus, in a situation of acidosis, the collecting duct compensates by secretion of protons into the lumen, whereas it can adapt to a situation of alkalosis by secreting bicarbonate into the lumen. The main feature of this mechanism is the specific directional insertion of the V-ATPase, which was already discussed in the previous section, and sAC appears to play a crucial role also in this system. Paunescu et al. [43] analyzed the localization of both the V-ATPase and sAC in the cortex of rat kidneys and found that these two proteins strictly colocalize in intercalated cells. In A-type IC, sAC staining is concentrated at the apical pole with much weaker staining at the basolateral pole. In B-type IC, sAC is present both at the apical and basolateral poles. The V-ATPase shows distinct overlap with sAC staining in both cell types. The authors demonstrated that the two proteins could be co-immunoprecipitated suggesting that they have a molecular interaction. The vectorial transport of protons by intercalated cells needs to be compensated by bicarbonate secretion at the opposite pole of the cell. Thus, in conjunction with the V-ATPase and sAC stainings, the authors also stained kidney sections for the chloride/bicarbonate exchangers AE1 and pendrin (an anion exchanger, the gene product of *SLC26A4*). It was found that in A-type IC AE1 is strongly concentrated on the basolateral side and thereby compensates for the apical proton secretion. On the other hand, in B-type cells, AE1 is absent, and pendrin is strictly localized on the apical pole, thereby compensating for the basolateral proton secretion. This specific localization pattern in A-type vs. B-type IC is not a static situation: depending on the intracellular pH, A-type IC can convert to B-type IC, and this conversion is achieved by rapid recycling of the V-ATPase via endosome retraction from the membrane and vesicular transport to the other pole. Given the strict colocalization of sAC and V-ATPase under various conditions as well as the molecular interaction of the two proteins, it is reasonable to assume that sAC plays a key role in this pH sensing mechanism.

### Embryonic fibroblasts

V-ATPase does not only play a role in proton secretion at the plasma membrane but also in lysosomal acidification. As mentioned in the previous section, V-ATPase undergoes extensive recycling between the plasma membrane and the endosomal compartment, and from the latter, it can also be recruited to the vacuolar compartments including lysosomes. Rahman et al. [48] investigated a potential interaction between sAC and V-ATPase in mouse embryonic fibroblasts (MEFs). They cultured these cells in the presence of FITC-dextran, which enters the cells by endocytosis and ends up in lysosomes. Since the fluorescence of FITC-dextran is pH-dependent, it could be used to monitor changes in lysosomal pH. Using this system, they found that incubation of the cells with the sAC inhibitor KH7 led to a significant increase of the pH by an apparent 0.5 pH unit. MEFs from sAC-KO mice [18] displayed this increased lysosomal pH already under basal conditions, but the addition of Sp-8-cpt-cAMP, a lipophilic and stable cAMP analog that activates PKA, brought the lysosomal pH back to the pH value observed and restored proper colocalization of V-ATPase with lysosomes in wild type cells. These data suggest that sAC regulates lysosomal pH through a sAC/PKA/V-ATPase axis. Interestingly, deficiency of sAC (in sAC-KO cells) also led to impaired lysosomal function; western blotting showed that LC3-II levels were increased, pointing to impaired autophagy and delivery to lysosomes where LC3-II is normally broken down.

### Osteoclasts

Osteoclasts are cells that function in the resorption of bone matrix during bone remodeling. These multinucleated cells are derived from the self-fusion of macrophages. This transformation is mainly mediated by the cytokine RANK-L, and tartrate-resistant acid phosphatase (TRAP) is the main marker of differentiated osteoclasts. During their attachment to the bone matrix, osteoclasts polarize and form a resorption lacuna in the space between its ruffled membrane and the bone matrix. Subsequent release of lysosomal enzymes and secretion of protons mediate the breakdown of the bone matrix. This acidification is mediated by the V-ATPase mentioned in the previous sections [59]. Geng et al. [21] studied this process and found that while the bicarbonate-free medium was permissive of osteoclast formation, high bicarbonate (24 mM) strongly reduced the transformation. In order to test whether sAC is involved in this process, they incubated the precursor cells with 2-hydroxyestradiol and observed a marked inhibition of multinucleation and TRAP expression. Western blotting demonstrated a sAC isoform of about 75 kD that could be knocked down by siRNA. siRNA-treated precursor cells also displayed strongly impaired differentiation into osteoclasts. As a measure of in situ osteoclast function in bone resorption, the authors incubated dissected mouse calvariae for 7 days in the presence of different HCO_3_^−^ concentrations (under 5% CO_2_) and in the presence and absence of 2-hydroxyestradiol and measured bone volume density. They observed a slight but significant increase in bone volume density in high bicarbonate (24 vs. 12 mM), and this increase was eliminated by simultaneous incubation with 2-hydroxyestradiol. These experiments suggest that the differentiation of macrophages into osteoclasts is inhibited by high bicarbonate, a regulation that appears to be mediated by sAC.

The osteoclast is an example of a cell that mediates vectorial transport of protons. In polarized cells (mostly epithelial cells), pH sensing and regulation are crucial, because proton secretion at one pole (usually the apical pole) needs to be tightly compensated by bicarbonate secretion at the other (basolateral) pole. In this process, carbonic anhydrase generates H + and HCO_3_^−^ from metabolic CO2 for contralateral extrusion. If this is not well regulated, these cells have a strongly aberrant intracellular pH and will go into apoptosis. In several epithelial cells, such as gastric parietal cells, ameloblasts (acid secretors involved in enamel formation of teeth), and osteoclasts, this basolateral compensation by bicarbonate extrusion is mediated by the chloride/bicarbonate exchanger AE2 encoded by the *Slc4a2* gene. Indeed, in *Slc4a2*^*−/−*^ mice, these cells display a high degree of dysfunction and apoptosis [26, 37, 50].

### Airway epithelial cells

Airway epithelial cells express motile cilia that are important for innate host defense by propelling mucus out of the airways. Several groups provided evidence for a role of cAMP in airway ciliary beating via activation of PKA [52, 53, 66]. Schmid et al. [54] studied whether sAC is involved in the regulation of ciliary beat frequency. They detected isoforms of sAC in airway epithelial cells with masses of 50, 75, and 190 kD and immunofluorescence studies suggested preferential localization of the 50 kD form in cilia. Using the anti-sAC antibody raised by Zippin et al. [68], they performed immunofluorescence and observed a striking concentration of sAC immunoreactivity at the apical pole of the cells. Analysis of ciliary beat frequency (CBF) revealed complex regulation by both intracellular pH (independent of bicarbonate) and by sAC-derived cAMP. The latter conclusion was based on the decrease in CBF by KH7, suggesting that sAC-derived cAMP increases CBF, probably via PKA. Subsequently, the same group confirmed and extended these results by showing that the 50 kD isoform, which was identified as an isoform translated from exon 5–12, increased in expression during ciliogenesis and was specifically targeted to the cilia and supported CBF. The 50 kD isoform lacks the first catalytic (C1) domain, but the authors hypothesized that the 50-kD isoform interacts with another C1-donating cyclase to become a fully active nucleotide cyclase [13].

## Speculation on the use of sAC inhibitors in pharmacological therapy of primary biliary cholangitis

Due to its diverse roles in cellular processes, sAC may also emerge as a potential therapeutic target for diseases. A potentially important example in this context may be primary biliary cholangitis (PBC). In this disease, which mostly affects middle-aged women, a progressive cholestasis occurs that is associated with the formation of autoantibodies against the mitochondrial pyruvate dehydrogenase complex and specific inflammation of the bile duct epithelial cells [14]. It is a matter of debate whether this autoimmunity is a primary or secondary aspect of the disease [14]. It has also been argued that several environmental factors may trigger the disease process [2, 58]. The crucial observation was made that in an early stage, PBC patients have decreased gene expression of *SLC4A2* in bile duct epithelial cells [44]. *SLC4A2* encodes the chloride/bicarbonate exchanger AE2. Mardones et al. [39] have shown that downregulation of *SLC4A2* leads to alkalinization of the cytosol accompanied by strong induction of sAC expression as well as bicarbonate-induced activation of the enzyme. This induces increased sensitivity of the cells towards toxic bile salts which are abundantly present in the bile duct [5, 11]. Hence, therapeutic inhibition of sAC activity may be beneficial to PBC patients. If the recently developed third-generation sAC inhibitors turn out to be safe, the efficacy of therapeutic administration to PBC patients should be explored.

## Conclusions

The important role of sAC in pH sensing and pH homeostasis inside cells has been documented in several different cell types. In some cases, evidence was based on experiments with the first-generation sAC inhibitor, KH7, which warrants caution on some of the data published. Thus far, most studies have investigated the role of sAC in acute sensing and regulation of intracellular pH and relatively little attention has been devoted to a potential role of sAC in long-term changes in intracellular pH and sAC-driven regulation of pH-regulating enzymes and transporters. All in all, literature data confirm the unique pH sensing properties of sAC and its influence on various important proton and bicarbonate translocating processes.

## Data Availability

This manuscript is a review of the available literature on the subject: “The role of soluble adenylyl cyclase in sensing and regulating intracellular pH”. Since this concerns only already published data from various research groups (including already published data of our own), availability of data and materials does not apply to this manuscript. No datasets were generated or analyzed during the current study.

## References

[1] Akbari A (2019). ADCY10 frameshift variant leading to severe recessive asthenozoospermia and segregating with absorptive hypercalciuria. Hum Reprod.

[2] Ala A (2006). Increased prevalence of primary biliary cirrhosis near Superfund toxic waste sites. Hepatology.

[3] Appukuttan A (2012). Type 10 adenylyl cyclase mediates mitochondrial Bax translocation and apoptosis of adult rat cardiomyocytes under simulated ischaemia/reperfusion. Cardiovasc Res.

[4] Balbach M (2023). On-demand male contraception via acute inhibition of soluble adenylyl cyclase. Nat Commun.

[5] Beuers U (2010). The biliary HCO(3)(-) umbrella: a unifying hypothesis on pathogenetic and therapeutic aspects of fibrosing cholangiopathies. Hepatology.

[6] Bizerra PFV (2024). Opposite regulation of glycogen metabolism by cAMP produced in the cytosol and at the plasma membrane. Biochim Biophys Acta Mol Cell Res.

[7] Braun DA (2016). Prevalence of monogenic causes in pediatric patients with nephrolithiasis or nephrocalcinosis. Clin J Am Soc Nephrol.

[8] Buck J (1999). Cytosolic adenylyl cyclase defines a unique signaling molecule in mammals. Proc Natl Acad Sci U S A.

[9] Buffone MG, Wertheimer EV, Visconti PE, Krapf D (2014). Central role of soluble adenylyl cyclase and cAMP in sperm physiology. Biochim Biophys Acta.

[10] Chaloupka JA (2006). Autoinhibitory regulation of soluble adenylyl cyclase. Mol Reprod Dev.

[11] Chang JC (2016). Soluble adenylyl cyclase regulates bile salt-induced apoptosis in human cholangiocytes. Hepatology.

[12] Chen Y (2000). Soluble adenylyl cyclase as an evolutionarily conserved bicarbonate sensor. Science.

[13] Chen X (2014). A soluble adenylyl cyclase form targets to axonemes and rescues beat regulation in soluble adenylyl cyclase knockout mice. Am J Respir Cell Mol Biol.

[14] Chen R, Tang R, Ma X, Gershwin ME (2022). Immunologic responses and the pathophysiology of primary biliary cholangitis. Clin Liver Dis.

[15] De Rasmo D, Panelli D, Sardanelli AM, Papa S (2008). cAMP-dependent protein kinase regulates the mitochondrial import of the nuclear encoded NDUFS4 subunit of complex I. Cell Signal.

[16] Demarco IA (2003). Involvement of a Na+/HCO-3 cotransporter in mouse sperm capacitation. J Biol Chem.

[17] Espejo MS (2020). The functional association between the sodium/bicarbonate cotransporter (NBC) and the soluble adenylyl cyclase (sAC) modulates cardiac contractility. Pflugers Arch.

[18] Esposito G (2004). Mice deficient for soluble adenylyl cyclase are infertile because of a severe sperm-motility defect. Proc Natl Acad Sci U S A.

[19] Forgac M (2007). Vacuolar ATPases: rotary proton pumps in physiology and pathophysiology. Nat Rev Mol Cell Biol.

[20] Geng W (2005). Cloning and characterization of the human soluble adenylyl cyclase. Am J Physiol Cell Physiol.

[21] Geng W (2009). Inhibition of osteoclast formation and function by bicarbonate: role of soluble adenylyl cyclase. J Cell Physiol.

[22] Ge Y et al (2024) Two novel heterozygous ADCY10 variants identified in Chinese pediatric patients with absorptive hypercalciuria: case report and literature review. J Genet 103 38258300

[23] Go S (2023). Cholangiocytes express an isoform of soluble adenylyl cyclase that is N-linked glycosylated and secreted in extracellular vesicles. Traffic.

[24] Halbritter J (2015). Fourteen monogenic genes account for 15% of nephrolithiasis/nephrocalcinosis. J Am Soc Nephrol.

[25] Hallows KR (2009). Regulation of epithelial Na+ transport by soluble adenylyl cyclase in kidney collecting duct cells. J Biol Chem.

[26] Jansen ID (2009). Ae2(a, b)-deficient mice exhibit osteopetrosis of long bones but not of calvaria. FASEB J.

[27] Kamenetsky M (2006). Molecular details of cAMP generation in mammalian cells: a tale of two systems. J Mol Biol.

[28] Kleinboelting S (2014). Crystal structures of human soluble adenylyl cyclase reveal mechanisms of catalysis and of its activation through bicarbonate. Proc Natl Acad Sci U S A.

[29] Kobayashi M, Buck J, Levin LR (2004). Conservation of functional domain structure in bicarbonate-regulated “soluble” adenylyl cyclases in bacteria and eukaryotes. Dev Genes Evol.

[30] Lark DS (2015). Protein kinase A governs oxidative phosphorylation kinetics and oxidant emitting potential at complex I. Front Physiol.

[31] Laudette M (2021). Cyclic AMP-binding protein Epac1 acts as a metabolic sensor to promote cardiomyocyte lipotoxicity. Cell Death Dis.

[32] Levine N, Marsh DJ (1971). Micropuncture studies of the electrochemical aspects of fluid and electrolyte transport in individual seminiferous tubules, the epididymis and the vas deferens in rats. J Physiol.

[33] Li Y (2021). Whole-exome sequencing of a cohort of infertile men reveals novel causative genes in teratozoospermia that are chiefly related to sperm head defects. Hum Reprod.

[34] Litvin TN, Kamenetsky M, Zarifyan A, Buck J, Levin LR (2003) Kinetic properties of “soluble” adenylyl cyclase. Synergism between calcium and bicarbonate. J Biol Chem 278(18):15922–15926 10.1074/jbc.M212475200 12609998

[35] Liu W (2023). Lactate modulates iron metabolism by binding soluble adenylyl cyclase. Cell Metab.

[36] Lv M (2022). Homozygous mutation in SLO3 leads to severe asthenoteratozoospermia due to acrosome hypoplasia and mitochondrial sheath malformations. Reprod Biol Endocrinol.

[37] Lyaruu DM (2008). The anion exchanger Ae2 is required for enamel maturation in mouse teeth. Matrix Biol.

[38] Lynch MJ, Hill EV, Houslay MD (2006). Intracellular targeting of phosphodiesterase-4 underpins compartmentalized cAMP signaling. Curr Top Dev Biol.

[39] Mardones P, Medina JF, Elferink RP (2008). Activation of cyclic AMP signaling in Ae2-deficient mouse fibroblasts. J Biol Chem.

[40] Maren TH (1967). Carbonic anhydrase: chemistry, physiology, and inhibition. Physiol Rev.

[41] Medina JF (2003). Anion exchanger 2 is essential for spermiogenesis in mice. Proc Natl Acad Sci U S A.

[42] Pastor-Soler N (2003). Bicarbonate-regulated adenylyl cyclase (sAC) is a sensor that regulates pH-dependent V-ATPase recycling. J Biol Chem.

[43] Paunescu TG (2008). Association of soluble adenylyl cyclase with the V-ATPase in renal epithelial cells. Am J Physiol Renal Physiol.

[44] Prieto J, Banales JM, Medina JF (2021). Primary biliary cholangitis: pathogenic mechanisms. Curr Opin Gastroenterol.

[45] Pozdniakova S, Ladilov Y (2018) Functional significance of the Adcy10-dependent intracellular cAMP compartments. J Cardiovasc Dev Dis **5**(2) 10.3390/jcdd5020029 PMC6023465 29751653

[46] Pucino V, Bombardieri M, Pitzalis C, Mauro C (2017). Lactate at the crossroads of metabolism, inflammation, and autoimmunity. Eur J Immunol.

[47] Puga Molina LC (2018). Molecular basis of human sperm capacitation. Front Cell Dev Biol.

[48] Rahman N (2016). Soluble adenylyl cyclase is essential for proper lysosomal acidification. J Gen Physiol.

[49] Ramos-Espiritu L (2016). Discovery of LRE1 as a specific and allosteric inhibitor of soluble adenylyl cyclase. Nat Chem Biol.

[50] Recalde S (2006). Inefficient chronic activation of parietal cells in Ae2a, b(-/-) mice. Am J Pathol.

[51] Reed BY (2002). Identification and characterization of a gene with base substitutions associated with the absorptive hypercalciuria phenotype and low spinal bone density. J Clin Endocrinol Metab.

[52] Sanderson MJ, Dirksen ER (1989). Mechanosensitive and beta-adrenergic control of the ciliary beat frequency of mammalian respiratory tract cells in culture. Am Rev Respir Dis.

[53] Schmid A (2006). Real-time analysis of cAMP-mediated regulation of ciliary motility in single primary human airway epithelial cells. J Cell Sci.

[54] Schmid A (2007). Soluble adenylyl cyclase is localized to cilia and contributes to ciliary beat frequency regulation via production of cAMP. J Gen Physiol.

[55] Schreiber M (1998). Slo3, a novel pH-sensitive K+ channel from mammalian spermatocytes. J Biol Chem.

[56] Shenroy AR, Visweswariah SS (2004). Class III nucleotide cyclases in bacteria and archaebacteria: lineage-specific expansion of adenylyl cyclases and a dearth of guanylyl cyclases. FEBS Lett.

[57] Strazzabosco M (2009). Differentially expressed adenylyl cyclase isoforms mediate secretory functions in cholangiocyte subpopulation. Hepatology.

[58] Tanaka A, Leung PS, Gershwin ME (2018). Environmental basis of primary biliary cholangitis. Exp Biol Med (Maywood).

[59] Teitelbaum SL (2000). Bone resorption by osteoclasts. Science.

[60] Turner TN (2019). Sex-based analysis of de novo variants in neurodevelopmental disorders. Am J Hum Genet.

[61] Visser L (2011). A comprehensive gene mutation screen in men with asthenozoospermia. Fertil Steril.

[62] Wang D (2007). A sperm-specific Na+/H+ exchanger (sNHE) is critical for expression and in vivo bicarbonate regulation of the soluble adenylyl cyclase (sAC). Proc Natl Acad Sci U S A.

[63] Wang C (2020). Use of whole-exome sequencing to identify a novel ADCY10 mutation in a patient with nephrolithiasis. Am J Transl Res.

[64] Windler F (2018). The solute carrier SLC9C1 is a Na(+)/H(+)-exchanger gated by an S4-type voltage-sensor and cyclic-nucleotide binding. Nat Commun.

[65] www.proteinatlas.org. Available from: https://www.proteinatlas.org/search/adcy10

[66] Wyatt TA, Spurzem JR, May K, Sisson JH (1998). Regulation of ciliary beat frequency by both PKA and PKG in bovine airway epithelial cells. Am J Physiol.

[67] Yusupova M (2023). Distinct cAMP signaling microdomains differentially regulate melanosomal pH and pigmentation. J Invest Dermatol.

[68] Zippin JH (2003). Compartmentalization of bicarbonate-sensitive adenylyl cyclase in distinct signaling microdomains. FASEB J.

